# Sodium Zirconium Cyclosilicate in CKD, Hyperkalemia, and Metabolic Acidosis: NEUTRALIZE Randomized Study

**DOI:** 10.34067/KID.0000000000000446

**Published:** 2024-04-16

**Authors:** Stephen R. Ash, Daniel Batlle, Jessica Kendrick, Yemisi Oluwatosin, Laura Kooienga, James M. Eudicone, Anna-Karin Sundin, Emily Guerrieri, Linda F. Fried

**Affiliations:** 1 Nephrology Department, Indiana University Health Arnett, Lafayette, Indiana; 2 Division of Nephrology and Hypertension, Department of Medicine, The Feinberg School of Medicine, Northwestern University, Chicago, Illinois; 3 Division of Renal Diseases and Hypertension, Department of Medicine, University of Colorado Anschutz Medical Campus, Aurora, Colorado; 4 Renal CVRM (US Medical), AstraZeneca, Wilmington, Delaware; 5 Colorado Kidney Care, Denver, Colorado; 6 BioPharmaceuticals Medical (Evidence), AstraZeneca, Wilmington, Delaware; 7 BioPharmaceuticals Medical (Evidence), AstraZeneca, Mölndal, Sweden; 8 Renal CVRM, AstraZeneca, Gaithersburg, Maryland; 9 Renal Section, Veterans Affairs Pittsburgh Healthcare System and Renal-Electrolyte Division, University of Pittsburgh, Pittsburgh, Pennsylvania

**Keywords:** acidosis, CKD

## Abstract

**Key Points:**

- Sodium zirconium cyclosilicate effectively lowers serum potassium and maintains normokalemia in patients with CKD with concomitant hyperkalemia and metabolic acidosis.
- Despite high screen failure and small sample size, a nominally significant increase in sHCO_3_^–^ was seen for sodium zirconium cyclosilicate versus placebo.
- Further studies on the basis of an appropriate cohort size may help validate the trend observed in sHCO_3_^–^ levels, supporting these clinically relevant findings.

**Background:**

Metabolic acidosis and hyperkalemia are common in CKD. A potential dual effect of sodium zirconium cyclosilicate (SZC), a selective binder of potassium in the gastrointestinal tract, on serum potassium (sK^+^) and serum bicarbonate (sHCO_3_^−^) was evaluated in patients with hyperkalemia and metabolic acidosis associated with CKD.

**Methods:**

In the NEUTRALIZE study (NCT04727528), non-dialysis patients with stage 3–5 CKD, hyperkalemia (sK^+^>5.0 to ≤5.9 mmol/L), and metabolic acidosis (sHCO_3_^−^ 16–20 mmol/L) received open-label SZC 10 g three times daily for ≤48 hours. Patients achieving normokalemia (sK^+^ 3.5–5.0 mmol/L) were randomized 1:1 to SZC 10 g or placebo daily for 4 weeks. The primary end point was patients (%) maintaining normokalemia at the end of treatment (EOT) without rescue. Secondary end points included mean change in sHCO_3_^−^ at EOT (day 29) and patients (%) with normokalemia with a ≥3-mmol/L increase in sHCO_3_^−^ without rescue.

**Results:**

Of 229 patients screened, 37 were randomized (SZC, *n*=17; placebo, *n*=20). High screen failure led to early study termination. At EOT, 88.2% (SZC) versus 20.0% (placebo) of patients maintained normokalemia (odds ratio, 56.2; *P* = 0.001). Low enrollment rendered secondary end point *P* values nominal. SZC treatment provided nominally significant increases in sHCO_3_^–^ versus placebo from day 15 onward. Patients with normokalemia with a ≥3-mmol/L increase in sHCO_3_^−^ without rescue were 35.3% (SZC) and 5.0% (placebo; *P* < 0.05). No new safety concerns were reported.

**Conclusions:**

SZC effectively lowered sK^+^ and maintained normokalemia, with nominally significant increases in sHCO_3_^–^ observed for SZC versus placebo.

## Visual Abstract

**Figure:**
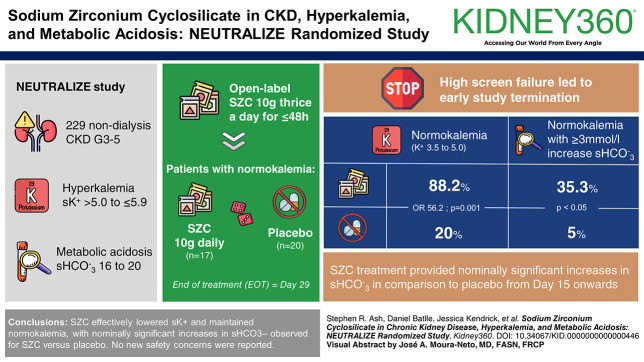

## Introduction

Metabolic acidosis or low serum bicarbonate (sHCO_3_^−^) is often one of the first recognized complications of renal failure^1^ and is associated with a broad spectrum of deleterious effects, including increased risk of CKD progression, bone demineralization, muscle catabolism, cardiovascular risk, and mortality.^2–7^

Available data demonstrate that correction of metabolic acidosis, for example, by administration of sodium bicarbonate, ameliorates adverse effects, including preserving kidney function and reducing risk of ESKD.^2,8–10^ In patients with CKD, guidelines, therefore, advocate that sHCO_3_^−^ concentrations <22 mmol/L should receive oral bicarbonate supplementation to maintain sHCO_3_^−^ within the normal range.^11^ Other studies, however, have reported only modest or inconclusive benefits.^9,12,13^

In patients with CKD, hyperkalemia (elevated serum potassium [sK^+^]) and metabolic acidosis frequently co-occur, linked by a complex relationship where hyperkalemia may cause, and be caused by, metabolic acidosis.^14^ Moreover, hyperkalemia, reduced eGFR, and reduced renal ammonium excretion are strong independent risk factors for development of metabolic acidosis, with each more than doubling acidotic risk.^15,16^ There is evidence for a close feedback/regulatory mechanism between potassium homeostasis and renal ammonia production where hyperkalemia can promote the development of metabolic acidosis through reduction of renal ammoniagenesis.^17–19^

Current treatment of acidosis (low sHCO_3_^−^) with sodium bicarbonate is limited by the associated high pill burden.^2^ Sodium zirconium cyclosilicate (SZC) is an oral nonabsorbed therapy approved for the treatment of hyperkalemia in adults that selectively captures potassium ions in exchange for sodium and hydrogen ions in the gastrointestinal lumen, increasing fecal excretion of potassium.^20,21^ Clinical trials of SZC showed significant reduction of sK^+^ levels and maintenance of normokalemia for up to 1 year with continued treatment,^22–24^ and *post hoc* analyses of phase 3 randomized trials further showed a dose-dependent increase in sHCO_3_^−^ with SZC.^22,24–26^ There is also clinical evidence that SZC has pleiotropic effects, for example, a renal protective benefit through improvement of acid–base balance.^23,27^

While the mechanism(s) here remain unclear, they likely involve augmentation of renal ammoniagenesis through correction of hyperkalemia^18,28^ or by binding and removal of ammonium by SZC in the gastrointestinal tract.^20^ SZC possesses a similar ionic binding site diameter for potassium and ammonium and, in aqueous solution, binds both cations *in vitro*.^20,26^ Recent *in vivo* experimental studies in a mouse model of CKD showed that SZC can sequester both ammonium and potassium in feces, demonstrating binding of these cations in the gastrointestinal tract.^29^

Given the potential dual benefits of SZC, and the clinical relevance of addressing both hyperkalemia and metabolic acidosis, the phase 3b NEUTRALIZE study evaluated the effect of SZC on sHCO_3_^−^ and sK^+^ in patients with hyperkalemia and metabolic acidosis associated with CKD.

## Methods

### Trial Design and Oversight

The NEUTRALIZE study was a phase 3b, randomized, double-blind, placebo-controlled, parallel-designed trial, conducted across 30 US sites from March 2021 to September 2022 (ClinicalTrials.gov identifier: NCT04727528; registered December 22, 2020). The study design, as reported previously,^30^ comprised a screening visit, a treatment period of approximately 4 weeks, and an off-treatment follow-up visit 1 week later.

During the open-label correction phase, patients received 10 g of SZC orally, three times daily for up to 48 hours. Patients achieving normokalemia (point-of-care test [POCT] potassium between 3.5 and 5.0 mmol/L inclusive) at day 2 (after 24 hours of open-label correction) entered the randomized phase. Patients not meeting normokalemia at day 2 continued in the open-label correction phase for an additional 24 hours. If normokalemia was achieved by day 3 (after up to a total of 48 hours open-label treatment), the patient entered the randomized maintenance phase; if not, treatment was discontinued, and the patient was withdrawn from the study. Those achieving normokalemia during the open-label correction phase were randomized 1:1 into the double-blind, maintenance phase to receive 10 g SZC or placebo once daily (starting dose) for 4 weeks, titrated at 1-week intervals for 2 weeks to achieve normokalemia (day 2 or 3 to day 29). Randomization was assigned through Interactive Response Technology/Randomization and Trial Supply Management and treatment dispensed by designated and trained site pharmacy staff. Investigators and pharmacists were blinded to allocated treatment. Point-of-care testing was performed using an Abbott i-STAT 1 portable blood analyzer or Piccolo Xpress analyzer.

Rescue therapy, administered for severe hyperkalemia (sK^+^ >6 mmol/L) or low sHCO_3_^−^ (≤15 mmol/L), was based on local practice standards and standard of care, respectively. Patients discontinued study treatment after rescue therapy. Patients receiving renin-angiotensin-aldosterone system inhibitors and sodium-glucose cotransporter 2 inhibitors at baseline (day 1) could be continued at the baseline dose; however, patients could not be started on renin-angiotensin-aldosterone system inhibitor or sodium-glucose cotransporter 2 inhibitor therapy during the trial.

The study was performed in compliance with the Declaration of Helsinki, Council for International Organizations of Medical Sciences International ethical guidelines, applicable International Council for Harmonisation and Good Clinical Practice guidelines, and relevant local regulations. The informed consent form, protocol, and all amendments were approved by an institutional review board. All participants provided written informed consent.

### Patients

Eligible adults (18 years and older) had stage 3−5 CKD, were not undergoing dialysis, and exhibited an eGFR of ≤59 ml/min per m^2^. In addition, they had to have a POCT potassium level of >5.0 to ≤5.9 mmol/L and a POCT bicarbonate level of 16−20 mmol/L inclusive, before their first SZC dose on study day 1. Detailed inclusion and exclusion criteria are provided in the Supplemental Materials and were previously reported by Ash *et al.*^30^

### Study Assessments

Study assessments were performed as previously reported.^30^ POCT measures of blood potassium and bicarbonate were used for the purposes of screening and evaluating need for dose adjustments during the open-label correction phase and were performed at prespecified time points during the randomized maintenance phase and at follow-up. Separate blood samples for efficacy analyses (including baseline measures) were drawn at the same time points during the randomized maintenance phase for analysis by the central laboratory. Safety was assessed in terms of adverse events (AEs), serious AEs (SAEs), AEs leading to treatment discontinuation, clinical laboratory safety measures, vital signs, electrocardiogram, and full physical examination during the open-label phase, the randomized maintenance phase, and through to follow-up.

### Study End Points

#### Primary End Point

The primary end point was proportion of patients with sK^+^ 3.5–5.0 mmol/L inclusive (*i.e*.*,* normokalemia) at the end of treatment (EOT) (day 29) without the need for rescue treatment of hyperkalemia at any point during the randomized maintenance phase.

#### Secondary End Points

The secondary end points were as follows: mean change in sHCO_3_^−^ from the start of treatment (day 1) to EOT (day 29), and the proportion of patients with increase in sHCO_3_^−^ of ≥2.0 or ≥3.0 mmol/L by day 29, without requirement of rescue therapy for metabolic acidosis; sHCO_3_^−^ level ≥22 mmol/L by EOT; sK^+^ level between 3.5 and 5.0 mmol/L, inclusive, at EOT and an increase in sHCO_3_^−^ of ≥3 mmol/L from baseline, without the need for rescue treatment of metabolic acidosis or hyperkalemia; and a sK^+^ level between 3.5 and 5.0 mmol/L, inclusive, and sHCO_3_^−^ ≥22 mmol/L at EOT, without the need for rescue interventions for metabolic acidosis or hyperkalemia. Patients requiring emergency interventions for low sHCO_3_^−^ levels anytime during the randomized maintenance phase were evaluated.

#### Planned Exploratory End Points

Mean change from baseline at EOT in serum chloride and aldosterone was the planned exploratory end point.

### Statistical Analysis

The sample size in the NEUTRALIZE study was guided by the observed proportion of patients with sHCO_3_^−^ between 16 and 20 mmol/L inclusive and a ≥3-mmol/L increase in sHCO_3_^−^ with SZC in previous studies.^24,25,31^ In powering for this secondary end point, it was anticipated to achieve approximately 80% power, assuming a 20% absolute difference in the proportion of responders (two-sided Fisher exact test; significance level 5%). Accordingly, it was planned to (approximately) screen 477 patients, enroll 148 patients into the open-label phase, and randomize 136 patients to the study intervention.

Analyses for the primary, secondary, and exploratory end points used the full analysis set, defined as all patients who achieved normokalemia (POCT potassium between 3.5 and 5.0 mmol/L inclusive) at the end of the open-label correction phase and who entered the randomized, placebo-controlled maintenance phase; patients were analyzed on an intent-to-treat basis by randomized treatment arm. Logistic regression analysis was used for the primary and secondary end points, with data presented as odds ratio (OR) and 95% confidence interval (CI); rescue treatment was the dependent variable and randomized treatment the independent variable. The secondary end point of mean sHCO_3_^−^ at EOT (day 29) was evaluated using analysis of covariance (randomized treatment as main effect and baseline value as covariate) and reported as least squares mean (LSM) with SEM and LSM (95% CI) for the between-group difference. The exploratory end points were similarly evaluated using analysis of covariance, with measures reported as mean (SD). Between-treatment group differences were reported as LSM (95% CI).

For the primary and secondary end points, sK^+^ and sHCO_3_^−^ measures made on day 22 were used if the measure on day 29 (EOT) was unavailable because of a missed visit or drop-out related to coronavirus disease 2019 and, if required, an approach to use POCT measures applied (last observation carried forward).

Safety analyses used the safety set: all patients who achieved normokalemia at the end of the open-label correction phase and received ≥1 dose of either SZC or placebo during the randomized placebo-controlled maintenance phase. No formal statistical analysis was performed, and findings are reported descriptively by treatment group.

All statistical analyses were performed using SAS version 9.3 or later (SAS Institute, Cary, NC).

## Results

### Patient Disposition and Baseline Characteristics

**Figure 1:**
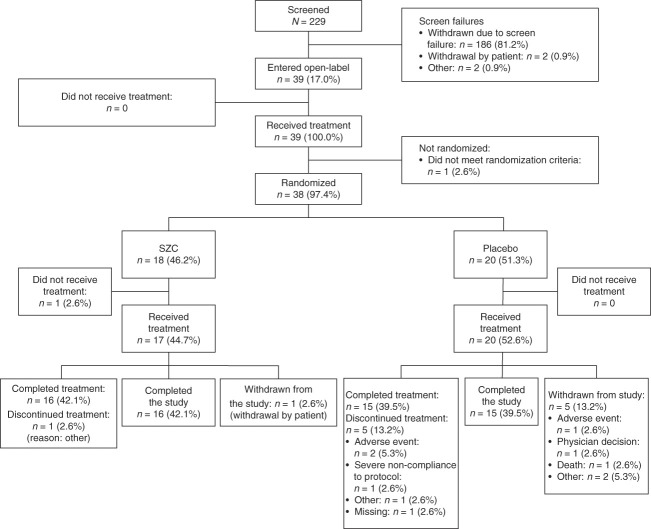
**Patient disposition.** *n*, number of subjects; SZC, sodium zirconium cyclosilicate.

A total of 229 patients were screened across 30 study sites. Owing to a higher-than-expected screen failure rate (83.0%; *n*=190), only 39 patients (17.0%) were enrolled and entered the open-label correction phase (Figure 1). The high screen failure rate led to early termination of the study (September 14, 2022), and the decision was unrelated to any safety concerns. Screen failure was primarily due to patients not meeting the inclusion criterion of POCT potassium >5.0 to ≤5.9 mmol/L and bicarbonate 16–20 mmol/L inclusive (>98% of cases). Other contributory factors included difficulty with repeated blood draws or effective venous catheterization and meeting of exclusion criteria. By the end of the open-label phase, one patient had not met the randomization criteria and another had been randomized but had not received treatment; thus, 37 patients were randomized and received treatment in the double-blind, placebo-controlled maintenance phase (SZC *n*=17; placebo *n*=20). Of these 37 patients, 31 completed treatment (SZC *n*=16; placebo *n*=15), with fewer patients discontinuing treatment with SZC (*n*=1) than with placebo (*n*=5).

Patient demographics and clinical characteristics are shown in Table 1. Overall, mean patient age was 63.3 years (48.6% were aged 18−64 years), and most of the patients were male (67.6%) and White (86.5%). Mean (SD) sK^+^ at baseline was 5.4 (0.4) mmol/L for SZC and 5.5 (0.4) mmol/L for placebo; for sHCO_3_^−^, these values were 16.1 (2.0) and 15.6 (2.6) mmol/L, respectively. Medical history was generally typical of the patient population studied; overall, 78.4% of patients had a reported history of hyperkalemia, 89.2% CKD (stage 3−5), 54.1% metabolic acidosis, and 91.9% hypertension. Notably, patients in the placebo group were slightly older (mean of 65.9 versus 60.3 years) with more at CKD stage 5 (*n*=6 versus *n*=1).

**Table 1:** Patient demographics and baseline characteristics (full analysis set)

Characteristic	SZC *n*=17	Placebo *n*=20	Total *n*=37
Age, yr, mean (SD)	60.3 (16.8)	65.9 (11.1)	63.3 (14.1)
**Age group, yr, *n* (%)**			
18–64	9 (52.9)	9 (45.0)	18 (48.6)
65–84	8 (47.1)	11 (55.0)	19 (51.4)
Sex, male, *n* (%)	13 (76.5)	12 (60.0)	25 (67.6)
**Race, *n* (%)**			
Black	3 (17.6)	1 (5.0)	4 (10.8)
Other	0	1 (5.0)	1 (2.7)
White	14 (82.4)	18 (90.0)	32 (86.5)
sK^+^, mmol/L, mean (SD)^a^	5.4 (0.4)	5.5 (0.4)	—
sHCO_3_^−^, mmol/L, mean (SD)	16.1 (2.0)	15.6 (2.6)	—
eGFR, ml/min per 1.73 m^2^, mean (SD)	23.2 (14.2)	20.5 (9.9)	—
Chronic heart failure, *n* (%)	0	0	0
Diabetes, *n* (%)	11 (64.7)	15 (75.0)	26 (70.3)

*n*, number of subjects; sHCO_3_^−^, serum bicarbonate; sK^+^, serum potassium; SZC, sodium zirconium cyclosilicate.

a Safety set randomized.

During the open-label correction phase, 97.4% of patients received ≥1 allowed concomitant medication, including angiotensin-converting enzyme inhibitors (56.4%) and angiotensin receptor blockers (17.9%). During the randomized maintenance phase, all patients received ≥1 allowed concomitant medication, with type and frequency generally consistent with the open-label phase.

### Primary End Point: Proportion of Responders

A significantly higher proportion of patients maintained normokalemia without the need for hyperkalemia rescue treatment at day 29 in the SZC group (88.2%; mean [SD] sK^+^ 4.6 [0.4] mmol/L) compared with the placebo group (20.0%; mean sK^+^ 5.3 [0.4] mmol/L); estimated OR (95% CI) was 56.2 (5.6 to 563.6; *P* = 0.001) (Table 2).

**Table 2:** Proportion of patients with serum potassium 3.5–5.0 mmol/L inclusive at day 29 (end of treatment) without the need for rescue treatment of hyperkalemia (full analysis set)

Treatment Arm	Subjects with Response, *n* (%)			Comparison between Groups	
	No Response	Response	Missing	OR (95% CI)	*P* Value
SZC (*n*=17)	1 (5.9)	15 (88.2)	1 (5.9)	56.2 (5.6 to 563.6)	0.001
Placebo (*n*=20)	15 (75.0)	4 (20.0)	1 (5.0)		

CI, confidence interval; *n*, number of subjects; OR, odds ratio; SZC, sodium zirconium cyclosilicate.

### Secondary and Exploratory End Points

Smaller than planned numbers of patients resulted in the study being underpowered to evaluate the secondary end points. As such, all *P* values reported are nominal.

#### Secondary

**Figure 2:**
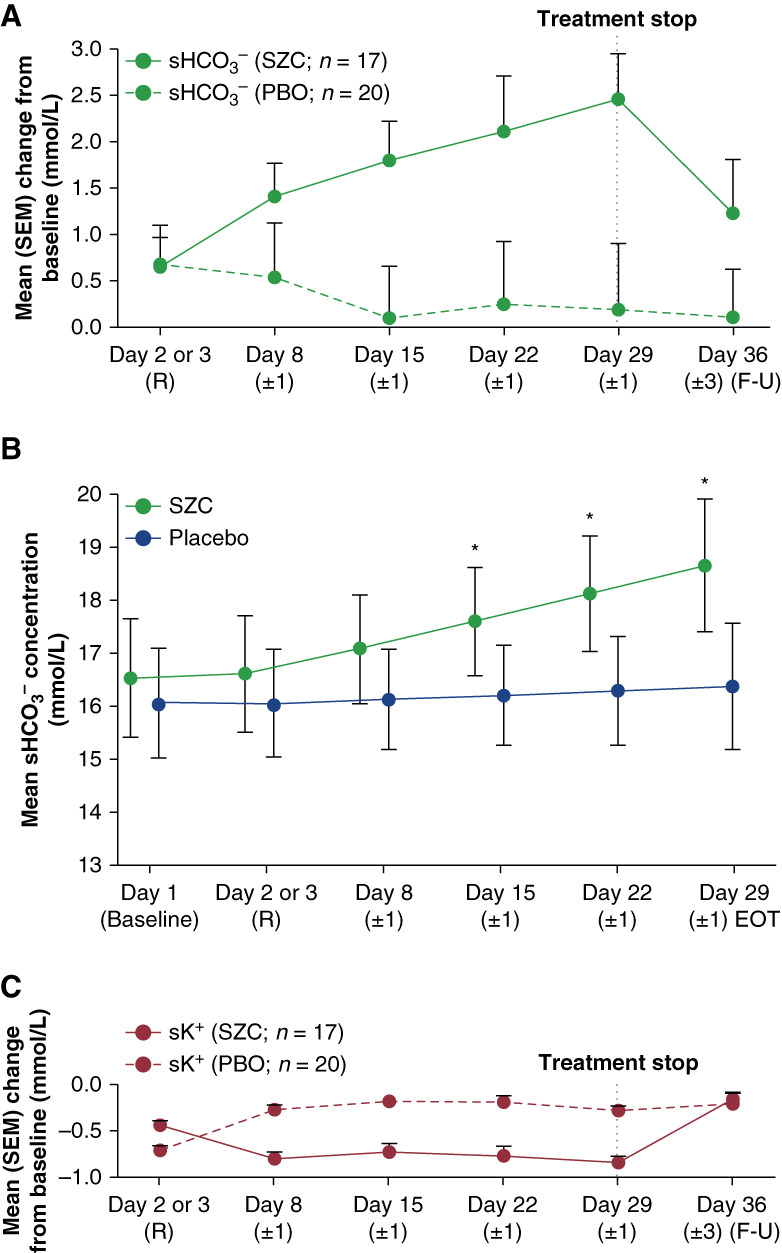
**Changes in concentration by visit.** (A) Change from baseline in sHCO_3_^–^ concentration. (B) Change in sHCO_3_^–^ concentration. (C) Change from baseline in sK^+^ concentration. *P ≤ 0.05. Full analysis set. Adjusted LSM (95% CI) estimates at each time point; mixed-model repeated-measures analysis. CI, confidence interval; EOT, end of treatment; F‐U, follow-up; LSM, least squares mean; PBO, placebo; R, randomization; sHCO_3_^–^, serum bicarbonate; sK^+^, serum potassium.

LSM (SEM) sHCO_3_^−^ concentration at EOT was 18.2 (0.6) mmol/L for SZC versus 16.5 (0.6) mmol/L for placebo. LSM (SEM) change from baseline in sHCO_3_^−^ at EOT was 2.1 (0.5) mmol/L for SZC versus 0.8 (0.7) mmol/L for placebo (between-group difference: 1.64; 95% CI, 0.00 to 3.29; *P* = 0.050). Over the randomized maintenance phase, treatment with SZC was associated with a trend toward an increase in mean sHCO_3_^–^ (Figure 2A) while a mixed-model repeated-measures analysis showed nominally significant increases in sHCO_3_^–^ concentration with SZC from day 15 onward (Figure 2B). SZC was further associated with a nominally significant decrease in sK^+^ concentration from baseline (Figure 2C).

The proportion of patients maintaining normokalemia with a ≥3-mmol/L increase in sHCO_3_^−^, without the need for rescue therapy for low sHCO_3_^−^ or hyperkalemia, was 35.3% for SZC versus 5.0% for placebo; estimated OR was 10.8 (*P* = 0.039) (Table 3). Only two patients in the SZC group had an increase of sHCO_3_^−^ to ≥22 mmol/L, which confounded interpretation for two secondary end points: sHCO_3_^−^ ≥22 mmol/L at EOT without the need for rescue for low sHCO_3_^−^ and sK^+^ within 3.5–5.0 mmol/L inclusive and sHCO_3_^−^ ≥22 mmol/L at EOT without the need for rescue for hyperkalemia or low sHCO_3_^−^. There were no measurable differences between the two treatment groups regarding the remaining secondary end points. No patient, in either treatment group, needed rescue therapy for low sHCO_3_^−^ during the randomized maintenance phase.

**Table 3:** Secondary end points (sodium zirconium cyclosilicate, *n*=17; placebo, *n*=20; full analysis set)

End Point	Treatment	Response, *n* (%)			Comparison Between Groups	
		No Response	Response	Missing	OR (95% CI)	*P* Value^a^
Increase in sHCO_3_^−^ ≥3 mmol/L at EOT *(no rescue for low sHCO*_*3*_^*−*^*)*	SZC	10 (58.8)	6 (35.3)	1 (5.9)	3.2 (0.6 to 15.8)	0.153
	Placebo	16 (80.0)	3 (15.0)	1 (5.0)		
sHCO_3_^−^ ≥22 mmol/L at EOT *(no rescue for low sHCO*_*3*_^*−*^*)*	SZC	14 (82.4)	2 (11.8)	1 (5.9)	NR	0.940
	Placebo	19 (95.0)	0	1 (5.0)		
Increase in sHCO_3_^−^ ≥2 mmol/L at EOT *(no rescue for low sHCO*_*3*_^*−*^*)*	SZC	8 (47.1)	8 (47.1)	1 (5.9)	2.2 (0.5 to 8.6)	0.271
	Placebo	13 (65.0)	6 (30.0)	1 (5.0)		
sK^+^ 3.5−5.0 mmol/L inclusive, and increase in sHCO_3_^−^ ≥3 mmol/L at EOT *(no rescue for HK or low sHCO*_*3*_^*−*^*)*	SZC	10 (58.8)	6 (35.3)	1 (5.9)	10.8 (1.1 to 102.8)	0.039
	Placebo	18 (90.0)	1 (5.0)	1 (5.0)		
sK^+^ 3.5−5.0 mmol/L inclusive, and sHCO_3_^−^ ≥22 mmol/L at EOT *(no rescue for HK or low sHCO*_*3*_^*−*^*)*	SZC	14 (82.4)	2 (11.8)	1 (5.9)	NR	0.940
	Placebo	19 (95.0)	0	1 (5.0)		

CI, confidence interval; EOT, end of treatment; HK, hyperkalemia; *n*, number of subjects; NR, not reported due to small sample numbers; OR, odds ratio; sHCO_3_^−^, serum bicarbonate; sK^+^, serum potassium; SZC, sodium zirconium cyclosilicate.

a *P* value calculated using Wald chi-squared test.

#### Exploratory

There was no evidence of any change or difference in serum chloride levels between treatment groups. Mean serum aldosterone levels at EOT were lower in patients who received SZC (42.7 pmol/L) compared with those who received placebo (182.0 pmol/L); between-group difference was −66.2 pmol/L (95% CI, −96.0 to −36.4; *P* < 0.001) (Supplemental Table 1).

#### Safety

The proportion of patients with an AE during the open-label correction phase was low, with three patients (7.7%) reporting three events, one of which (mild hyperglycemia) was considered by the investigator as possibly treatment related. There were no deaths, no SAEs, no AEs leading to dose modification or interruption, and no AEs leading to discontinuation of either treatment during the open-label correction phase.

In the randomized maintenance phase, four patients with SZC (eight events; 23.5%) and nine patients with placebo (24 events; 45.0%) had an AE (Table 4). SAEs were reported by 1 patient (5.9%) in the SZC group (hypertension) and 2 patients (10.0%) in the placebo group (AKI and hypervolemia).

**Table 4:** Adverse events in any category (safety set)

Event	SZC, *n* (%) *n*=17	Placebo, *n* (%) *n*=20
Any AE	4 (23.5)	9 (45.0)
Any AE possibly related to treatment	0	2 (10.0)
Any AE with outcome=death	0	0
Any SAE (including events with outcome=death)	1 (5.9)	2 (10.0)
Any AE leading to treatment discontinuation	0	3 (15.0)
Any SAE leading to treatment discontinuation	0	1 (5.0)
Any AE leading to dose interruption	0	1 (5.0)
Any AE leading to dose reduction	0	0
Any AE leading to study discontinuation	0	2 (10.0)

Included adverse events that occurred during the randomized maintenance phase and up to 7 days after discontinuation of investigational product. AE, adverse event; *n*, number of subjects; SAE, serious adverse event; SZC, sodium zirconium cyclosilicate.

No serious adverse events occurred during the open-label phase.

The most common AEs reported by >1 patient (≥10%) were hyperkalemia and nausea in the placebo group; most (preferred term) AEs were reported only once for SZC. No AE was considered related to SZC, and no AE led to dose modification/interruption or discontinuation of SZC. No deaths occurred during the study period. The incidence of edema-related AEs was low in both treatment groups (SZC, *n*=1 patient; placebo, *n*=2 patients); all were of mild intensity and not considered related to study treatment by the investigator. During the randomized maintenance phase, there were no clinical chemistry-related AEs in the SZC group (compared with seven in the placebo group). Furthermore, there were no clinically relevant trends in clinical chemistry measures, vital signs, or electrocardiogram values for either treatment group. Two patients in the SZC group developed hypertension, one serious but not considered related to SZC by the investigator.

#### Clinical Laboratory Measures

No clinically relevant trends were apparent in mean changes from baseline in clinical laboratory measures over time for either treatment group, with the exception of sK^+^, sHCO_3_^−^, and serum urea nitrogen. Median levels of serum urea nitrogen were similar in both treatment groups at baseline (SZC 21.4 mmol/L, *n*=17; placebo 20.7 mmol/L, *n*=17); at EOT, median change from baseline was −3.6 mmol/L for SZC compared with 0 mmol/L for placebo.

## Discussion

NEUTRALIZE is the first study designed to evaluate the effect of an antihyperkalemia therapy on both sHCO_3_^−^ and sK^+^ in patients with CKD and co-occurrence of hyperkalemia and metabolic acidosis. NEUTRALIZE met its primary end point, demonstrating that continued treatment with SZC lowered sK^+^ and maintained normokalemia for up to 4 weeks in CKD patients with hyperkalemia and metabolic acidosis, findings consistent with previous studies of SZC in outpatients with hyperkalemia.^24,25^ Safety and tolerability were similarly consistent with the established safety profile of SZC, the incidence of AEs being low and mild in intensity.

Data analysis was affected by a high screen failure rate, leading to a small sample size and early termination of the study. The primary reason for screen failure was sK^+^ and/or sHCO_3_^−^ levels falling outside the range for inclusion before the first treatment dose (>98% of cases). Strengths of the NEUTRALIZE study design were the inclusion of only patients with low sHCO_3_^−^ at baseline, control for the use of potentially confounding agents, such as sodium bicarbonate, and that patients were not allowed to have their sodium bicarbonate therapy changed. Further studies might benefit from a less restrictive inclusion limit for sHCO_3_^−^ because NEUTRALIZE inclusion criteria did not cover the full range associated with metabolic acidosis.

NEUTRALIZE not only met its primary end point of achieving normokalemia but also showed nominally significant increases in sHCO_3_^−^ with SZC, with changes from baseline being generally consistent with previously reported data from SZC clinical trials.^24,32^ For the exploratory end points, the observed reduction in serum aldosterone at EOT for SZC was consistent both with the HARMONIZE study in outpatients with hyperkalemia treated with SZC for 29 days^25^ and with trials reporting reduced aldosterone alongside increases in sHCO_3_^−^.^33^

Notably, the observed reduction in serum urea nitrogen at EOT with SZC in the NEUTRALIZE study is consistent with recent path analyses showing that an increase in sHCO_3_^−^ with SZC is associated with a decrease in serum urea rather than with changes in urine pH.^26^ In this instance, serum urea and urine pH were used as proxies of gastrointestinal ammonium trapping and renal ammoniagenesis in the absence of measures of fecal and urinary ammonium, respectively.^26^ This supports the hypothesis that SZC raises sHCO_3_^−^ by trapping ammonium in the gastrointestinal tract, mostly in exchange for sodium,^20,26^ as shown recently in a mouse model of CKD demonstrating *in vivo* sequestration of ammonium in the gastrointestinal tract by SZC.^29^

In summary, SZC effectively lowers sK^+^ and maintains normokalemia with continued treatment for up to 4 weeks in patients with CKD with concomitant hyperkalemia and metabolic acidosis. In addition, SZC was associated with nominally significant increases in sHCO_3_^−^ compared with placebo. Further studies, on the basis of appropriate cohort size, may help validate the trend observed in sHCO_3_^−^ levels, supporting these clinically relevant findings.

## Supplementary Material

## Data Availability

All data are included in the manuscript and/or supporting information. Partial restrictions to the data and/or materials apply. “Data underlying the findings described in this manuscript may be requested in accordance with AstraZeneca’s data sharing policy described at https://astrazenecagrouptrials.pharmacm.com/ST/Submission/Disclosure. The AstraZeneca Group of Companies allows researchers to submit a request to access anonymized patient level clinical data, aggregate clinical or genomics data (when available), and anonymized clinical study reports through the Vivli web-based data request platform.”

## Supplemental Material

Inclusion criteria

Exclusion criteria

Supplemental Table 1. Exploratory end points (change from baseline at the end of treatment; full analysis set).
