# Supplementary material for: Sodium Zirconium Cyclosilicate in CKD, Hyperkalemia, and Metabolic Acidosis: NEUTRALIZE Randomized Study
Source: Kidney360. 2024 Apr 16;5(6):812–20. doi: 10.34067/KID.0000000000000446 (PMC11219110; doi:10.34067/KID.0000000000000446)
Supplement: Supplementary file 1 [file kidney360-5-812-s001.pdf]

ASN American Society of Nephrology | JASN Journal of the American Society of Nephrology | KIDNEY360 Accessing Our World From Every Angle | CJASN Clinical Journal of the American Society of Nephrology

ASN Journal Disclosure Form

As per ASN journal policy, I have disclosed any financial relationship or commitment held by myself and/or my spouse/partner in the past 36 months as included below. I have listed my Current Employer below to indicate there is a relationship requiring disclosure. If no relationship exists, my Current Employer is not listed.

S. Ash reports the following:
Employer: HemoCleanse Technologies LLC and Ash Access Technology; Consultancy: I was a paid consultant for NextKidney of the Netherlands in 2023, to formulate plans for a clinical trial of a new dialysis system.; Ownership Interest: I am sole owner of HemoCleanse Technologies LLC, having bought this subsidiary from HemoCleanse, Inc in 2015. I also own stock in Ash Access Technology, Inc. Neither of these companies produce or market medical devices or drugs. HemoCleanse Technologies has not yet licensed any technologies to other companies. I own a very small amount of stock in the VascAlert company (dialysis access monitoring) and Emano, Inc (urinary flow rate through audio analysis).; Patents or Royalties: Merit Medical pays royalties on a dialysis catheter to Ash Access Technology, and this royalty is increasing net worth of this company. The company may eventually distribute this value to the company shareholders.; and Advisory or Leadership Role: I am on the Editorial Board of J. of Vascular Research, Hemodialysis International, Peritoneal Dialysis International, and ASAIO Journal.   This position is not paid.

I understand that the information above will be published within the journal article, if accepted, and that failure to comply and/or to accurately and completely report the potential financial conflicts of interest could lead to the following: 1) Prior to publication, article rejection, or 2) Post-publication, sanctions ranging from, but not limited to, issuing a correction, reporting the inaccurate information to the authors' institution, banning authors from submitting work to ASN journals for varying lengths of time, and/or retraction of the published work.

Name: Stephen R. Ash
Manuscript ID: K360-2024-000082
Manuscript Title: Sodium Zirconium Cyclosilicate in Chronic Kidney Disease, Hyperkalemia, and Metabolic Acidosis: NEUTRALIZE Randomized Study
Date of Completion: March 15, 2024
Disclosure Updated Date: March 15, 2024

ASN
American Society of Nephrology
JASN
Journal of the American Society of Nephrology
KIDNEY360
Accessing Our World From Every Angle
CJASN
Clinical Journal of the American Society of Nephrology

ASN Journal Disclosure Form

As per ASN journal policy, I have disclosed any financial relationship or commitment held by myself and/or my spouse/partner in the past 36 months as included below. I have listed my Current Employer below to indicate there is a relationship requiring disclosure. If no relationship exists, my Current Employer is not listed.

D. Batlle reports the following:
Employer: Northwestern University Feinberg School of Medicine; Consultancy: Renibus, Astra Zeneca and Advicenne.; Ownership Interest: Angiotensin Therapeutics Inc.; Research Funding: NIDDK; Feinberg Foundation; Astra Zeneca; Honoraria: Renibus, Advicenne.; and Patents or Royalties: Founder and main owner of Angiotensin Therapeutics Inc. No royalties or income at this time.

I understand that the information above will be published within the journal article, if accepted, and that failure to comply and/or to accurately and completely report the potential financial conflicts of interest could lead to the following: 1) Prior to publication, article rejection, or 2) Post-publication, sanctions ranging from, but not limited to, issuing a correction, reporting the inaccurate information to the authors' institution, banning authors from submitting work to ASN journals for varying lengths of time, and/or retraction of the published work.

Name: Daniel Batlle
Manuscript ID: K360-2024-000082R1
Manuscript Title: "Sodium Zirconium Cyclosilicate in Chronic Kidney Disease, Hyperkalemia, and Metabolic Acidosis: NEUTRALIZE Randomized Study
Date of Completion: March 29, 2024
Disclosure Updated Date: January 22, 2024

ASN American Society of Nephrology
JASN Journal of the American Society of Nephrology
KIDNEY360 Accessing Our World From Every Angle
CJASN Clinical Journal of the American Society of Nephrology

ASN Journal Disclosure Form

As per ASN journal policy, I have disclosed any financial relationship or commitment held by myself and/or my spouse/partner in the past 36 months as included below. I have listed my Current Employer below to indicate there is a relationship requiring disclosure. If no relationship exists, my Current Employer is not listed.

J. Eudicone reports the following:
Employer: Astrazeneca; and Ownership Interest: Astrazeneca.

I understand that the information above will be published within the journal article, if accepted, and that failure to comply and/or to accurately and completely report the potential financial conflicts of interest could lead to the following: 1) Prior to publication, article rejection, or 2) Post-publication, sanctions ranging from, but not limited to, issuing a correction, reporting the inaccurate information to the authors' institution, banning authors from submitting work to ASN journals for varying lengths of time, and/or retraction of the published work.

Name: James M. Eudicone
Manuscript ID: K360-2024-000082R1
Manuscript Title: Sodium Zirconium Cyclosilicate in Chronic Kidney Disease, Hyperkalemia, and Metabolic Acidosis: NEUTRALIZE Randomized Study
Date of Completion: March 18, 2024
Disclosure Updated Date: March 15, 2024

ASN American Society of Nephrology | JASN Journal of the American Society of Nephrology | KIDNEY360 Accessing Our World From Every Angle | CJASN Clinical Journal of the American Society of Nephrology

ASN Journal Disclosure Form

As per ASN journal policy, I have disclosed any financial relationship or commitment held by myself and/or my spouse/partner in the past 36 months as included below. I have listed my Current Employer below to indicate there is a relationship requiring disclosure. If no relationship exists, my Current Employer is not listed.

L. Fried reports the following:
Employer: VA Pittsburgh Healthcare System; Consultancy: Novonordisk, Regeneron: Data Safety Monitoring boards; Ownership Interest: Stock ownership:; Amgen; Dow, 3M; and Research Funding: Astra Zeneca.

I understand that the information above will be published within the journal article, if accepted, and that failure to comply and/or to accurately and completely report the potential financial conflicts of interest could lead to the following: 1) Prior to publication, article rejection, or 2) Post-publication, sanctions ranging from, but not limited to, issuing a correction, reporting the inaccurate information to the authors' institution, banning authors from submitting work to ASN journals for varying lengths of time, and/or retraction of the published work.

Name: Linda F. Fried
Manuscript ID: K360-2024-000082R1
Manuscript Title: Sodium Zirconium Cyclosilicate in Chronic Kidney Disease, Hyperkalemia, and Metabolic Acidosis: NEUTRALIZE Randomized Study
Date of Completion: March 26, 2024
Disclosure Updated Date: March 26, 2024

ASN American Society of Nephrology
JASN Journal of the American Society of Nephrology
KIDNEY360 Accessing Our World From Every Angle
CJASN Clinical Journal of the American Society of Nephrology

ASN Journal Disclosure Form

As per ASN journal policy, I have disclosed any financial relationship or commitment held by myself and/or my spouse/partner in the past 36 months as included below. I have listed my Current Employer below to indicate there is a relationship requiring disclosure. If no relationship exists, my Current Employer is not listed.

E. Guerrieri reports the following:
Employer: AstraZeneca

I understand that the information above will be published within the journal article, if accepted, and that failure to comply and/or to accurately and completely report the potential financial conflicts of interest could lead to the following: 1) Prior to publication, article rejection, or 2) Post-publication, sanctions ranging from, but not limited to, issuing a correction, reporting the inaccurate information to the authors' institution, banning authors from submitting work to ASN journals for varying lengths of time, and/or retraction of the published work.

Name: Emily Guerrieri
Manuscript ID: K360-2024-000082R1
Manuscript Title: Sodium Zirconium Cyclosilicate in Chronic Kidney Disease, Hyperkalemia, and Metabolic Acidosis: NEUTRALIZE Randomized Study
Date of Completion: March 30, 2024
Disclosure Updated Date: May 12, 2023

ASN
American Society of Nephrology
JASN
Journal of the American Society of Nephrology
KIDNEY360
Accessing Our World From Every Angle
CJASN
Clinical Journal of the American Society of Nephrology

ASN Journal Disclosure Form

As per ASN journal policy, I have disclosed any financial relationship or commitment held by myself and/or my spouse/partner in the past 36 months as included below. I have listed my Current Employer below to indicate there is a relationship requiring disclosure. If no relationship exists, my Current Employer is not listed.

J. Kendrick reports the following:
Employer: University of Colorado Denver; Research Funding: Fresenius Medical Care Renal Therapies Group; Bayer; Pathalys; Honoraria: Pathalys Pharma; and Advisory or Leadership Role: Velphoro Medical Advisory Board; AMGEN Medical Advisory Board; Astra Zeneca Medical Advisory Committee; Pathalys Pharma Inc.

I understand that the information above will be published within the journal article, if accepted, and that failure to comply and/or to accurately and completely report the potential financial conflicts of interest could lead to the following: 1) Prior to publication, article rejection, or 2) Post-publication, sanctions ranging from, but not limited to, issuing a correction, reporting the inaccurate information to the authors' institution, banning authors from submitting work to ASN journals for varying lengths of time, and/or retraction of the published work.

Name: Jessica B. Kendrick
Manuscript ID: K360-2024-000082R1
Manuscript Title: Sodium Zirconium Cyclosilicate in Chronic Kidney Disease, Hyperkalemia, and Metabolic Acidosis: NEUTRALIZE Randomized Study
Date of Completion: March 15, 2024
Disclosure Updated Date: January 12, 2024

ASN
American Society of Nephrology
JASN
Journal of the American Society of Nephrology
KIDNEY360
Accessing Our World From Every Angle
CJASN
Clinical Journal of the American Society of Nephrology

ASN Journal Disclosure Form

As per ASN journal policy, I have disclosed any financial relationship or commitment held by myself and/or my spouse/partner in the past 36 months as included below. I have listed my Current Employer below to indicate there is a relationship requiring disclosure. If no relationship exists, my Current Employer is not listed.

L. Kooienga reports the following:
Employer: Colorado Kidney Care; and Research Funding: Akebia Therapeutics; Ardelyx ; AstraZeneca; Boehringer Ingelheim; Cara Therapeutics; Chinook Therapeutics; CSL Behring, FibroGen Inc. ; GlaxoSmithKline Pharmaceuticals; Goldfinch Bio; Omeros; Otsuka Pharmaceutical; Reata Pharmaceuticals ; Travere Therapeutics ; Tricida Inc.; Vera Therapeutics; Visterra;.

I understand that the information above will be published within the journal article, if accepted, and that failure to comply and/or to accurately and completely report the potential financial conflicts of interest could lead to the following: 1) Prior to publication, article rejection, or 2) Post-publication, sanctions ranging from, but not limited to, issuing a correction, reporting the inaccurate information to the authors' institution, banning authors from submitting work to ASN journals for varying lengths of time, and/or retraction of the published work.

Name: Laura Kooienga
Manuscript ID: K360-2024-000082R1
Manuscript Title: Sodium Zirconium Cyclosilicate in Chronic Kidney Disease, Hyperkalemia, and Metabolic Acidosis: NEUTRALIZE Randomized Study
Date of Completion: March 17, 2024
Disclosure Updated Date: March 17, 2024

ASN American Society of Nephrology | JASN Journal of the American Society of Nephrology | KIDNEY360 Accessing Our World From Every Angle | CJASN Clinical Journal of the American Society of Nephrology

ASN Journal Disclosure Form

As per ASN journal policy, I have disclosed any financial relationship or commitment held by myself and/or my spouse/partner in the past 36 months as included below. I have listed my Current Employer below to indicate there is a relationship requiring disclosure. If no relationship exists, my Current Employer is not listed.

Y. Oluwatosin reports the following:
Employer: Currently in Transition; Previously AstraZeneca; and Ownership Interest: AstraZeneca.

I understand that the information above will be published within the journal article, if accepted, and that failure to comply and/or to accurately and completely report the potential financial conflicts of interest could lead to the following: 1) Prior to publication, article rejection, or 2) Post-publication, sanctions ranging from, but not limited to, issuing a correction, reporting the inaccurate information to the authors' institution, banning authors from submitting work to ASN journals for varying lengths of time, and/or retraction of the published work.

Name: Yemisi Oluwatosin
Manuscript ID: K360-2024-000082R1
Manuscript Title: Sodium Zirconium Cyclosilicate in Chronic Kidney Disease, Hyperkalemia, and Metabolic Acidosis: NEUTRALIZE Randomized Study
Date of Completion: March 26, 2024
Disclosure Updated Date: May 15, 2023

ASN American Society of Nephrology | JASN Journal of the American Society of Nephrology | KIDNEY360 Accessing Our World From Every Angle | CJASN Clinical Journal of the American Society of Nephrology

ASN Journal Disclosure Form

As per ASN journal policy, I have disclosed any financial relationship or commitment held by myself and/or my spouse/partner in the past 36 months as included below. I have listed my Current Employer below to indicate there is a relationship requiring disclosure. If no relationship exists, my Current Employer is not listed.

A. Sundin reports the following:
Employer: AstraZeneca; and Ownership Interest: AstraZeneca.

I understand that the information above will be published within the journal article, if accepted, and that failure to comply and/or to accurately and completely report the potential financial conflicts of interest could lead to the following: 1) Prior to publication, article rejection, or 2) Post-publication, sanctions ranging from, but not limited to, issuing a correction, reporting the inaccurate information to the authors' institution, banning authors from submitting work to ASN journals for varying lengths of time, and/or retraction of the published work.

Name: Anna-Karin Sundin
Manuscript ID: K360-2024-000082R1
Manuscript Title: Sodium Zirconium Cyclosilicate in Chronic Kidney Disease, Hyperkalemia, and Metabolic Acidosis: NEUTRALIZE Randomized Study
Date of Completion: March 15, 2024
Disclosure Updated Date: March 15, 2024
