# Supplementary material for: Sodium Zirconium Cyclosilicate in CKD, Hyperkalemia, and Metabolic Acidosis: NEUTRALIZE Randomized Study
Source: Kidney360. 2024 Apr 16;5(6):812–20. doi: 10.34067/KID.0000000000000446 (PMC11219110; doi:10.34067/KID.0000000000000446)
Supplement: Supplementary file 2 [file kidney360-5-812-s002.pdf]

Supplementary material for: Sodium Zirconium Cyclosilicate in Chronic Kidney Disease, Hyperkalemia, and Metabolic Acidosis: NEUTRALIZE Randomized Study

Stephen R. Ash, MD[1†] Daniel Batlle, MD[2] Jessica Kendrick, MD[3] Yemisi Oluwatosin, PhD[4†] Laura Kooienga, MD[5] James M. Eudicone, MS, MBA[6] Anna-Karin Sundin, BSc[7] Emily Guerrieri, PharmD[8] and Linda F. Fried, MD[9]

[1]*Nephrology Department, Indiana University Health Arnett, Lafayette, Indiana*

[2]*Division of Nephrology and Hypertension, Department of Medicine, The Feinberg School of Medicine, Northwestern University, Chicago, Illinois*

[3]*Division of Renal Diseases and Hypertension, Department of Medicine, University of Colorado Anschutz Medical Campus, Aurora, Colorado*

[4]*Renal CVRM (US Medical), AstraZeneca, Wilmington, Delaware*

[5]*Colorado Kidney Care, Denver, Colorado*

[6]*BioPharmaceuticals Medical (Evidence), AstraZeneca, Wilmington, Delaware*

[7]*BioPharmaceuticals Medical (Evidence), AstraZeneca, Mölndal, Sweden*

[8]*Renal CVRM, AstraZeneca, Gaithersburg, Maryland*

[9]*Renal Section, Veterans Affairs Pittsburgh Healthcare System and Renal-Electrolyte Division, University of Pittsburgh, Pittsburgh, Pennsylvania*

[†]*Affiliation at time of study.*

Correspondence: Dr. Stephen R. Ash, HemoCleanse Technologies, LLC and Ash Access Technology, Inc., 3601 Sagamore Parkway North, Suite B Lafayette, Indiana 47904, USA. Tel. +1 765 427 7007. Email, sash@hemocleanse.com.

Contents

Inclusion criteria 3

Exclusion criteria 4

Supplementary Table S1. Exploratory endpoints (change from baseline at EOT; full analysis set).. 7

Inclusion criteria

- Adult aged ≥18 years.
- Stage 3–5 CKD and not on dialysis, with an estimated glomerular filtration rate (eGFR) ≤59 ml/min/m^2 based on the Chronic Kidney Disease Epidemiology Collaboration (CKD-EPI) equation.
- Point of care test (POCT) potassium >5 mmol/l to ≤5.9 mmol/l and POCT bicarbonate 16–20 mmol/l inclusive, prior to the first sodium zirconium cyclosilicate (SZC) dose on study Day 1.
- Ability to have repeated blood draws or effective venous catheterization.
- Contraceptive use by either sex consistent with local regulations regarding the methods of contraception for those participating in clinical studies.
- Negative urine pregnancy test at screening for females of childbearing potential.
- Female participants 1 year post-menopausal, surgically sterile, or using one highly effective form of birth control (failure rate of <1% per year when used consistently and correctly).
- Stable on chosen method of birth control for ≥3 months before entering the study and willing to remain on the birth control until 12 weeks after the last dose.
- Capable of giving signed informed consent; including compliance with requirements and restrictions listed in the informed consent form.
- Provision of signed and dated written Optional Genetic Research Information informed consent prior to collection of samples for optional genetic research that

supports the Genomic Initiative (genomic blood sample not mandatory).

Exclusion criteria

- Presence of pseudohyperkalemia.
- Dialysis requirement or anticipated by the investigator to require dialysis therapy within 1 month, history of renal transplant, or life expectancy <3 months.
- Cardiac arrhythmias requiring immediate treatment.
- Active or suspected diabetic ketoacidosis.
- POCT bicarbonate low enough to need emergency intervention or treatment as judged by the investigator.
- Acute/chronic worsening of renal function (e.g., ≥30% decline in eGFR) in the 3 months before screening.
- Current acute decompensated heart failure (HF), hospitalization due to decompensated HF within 4 weeks prior to screening, or myocardial infarction, unstable angina, stroke, or transient ischemic attack within 12 weeks prior to screening.
- Coronary revascularization (percutaneous coronary intervention or coronary artery bypass grafting) or valvular repair/replacement within 12 weeks prior to screening or planned to undergo any of these operations.
- Symptomatic hypotension.
- Current exacerbation of chronic obstructive pulmonary disease (COPD)/asthma or hospitalization due to exacerbation of COPD/asthma within 4 weeks of screening.

- Severe constipation, bowel obstruction or impaction, including abnormal postoperative bowel motility disorders.
- Active malignancy requiring treatment.
- History of QT prolongation associated with other medications that required discontinuation of that medication.
- Congenital long QT syndrome.
- Symptomatic or uncontrolled atrial fibrillation despite treatment, or asymptomatic sustained ventricular tachycardia. Patients with atrial fibrillation controlled by medication were permitted.
- QTcF (QT interval corrected by the Fridericia method) >550 msec.
- Active treatment (within 7 days prior to screening) with SZC, sodium bicarbonate, sodium polystyrene sulfonate, lactulose, or patiromer.
- Participation in another clinical study with an investigational product administered in the past month.
- Known hypersensitivity to SZC.
- Involvement in the planning and/or conduct of the study (applies to both AstraZeneca staff and/or staff at the study site).
- Severe physically or mentally incapacitation that, in the opinion of the investigator, means the patient is unable to perform the tasks associated with the protocol.
- Previous enrollment in the present study.

- Currently pregnant (confirmed with positive pregnancy test) or breastfeeding.

- Evidence of coronavirus disease 2019 within 2 weeks prior to screening.

Supplementary Table S1. Exploratory endpoints (change from baseline at EOT; full analysis set)

	SZC/placebo, *n*	Value at BL, SZC/placebo, mean (SD)	Value at EOT, SZC/placebo, mean (SD)	Comparison between groups LSM (95% CI)	*P-value*
Serum chloride, mmol/l	16 / 14	107.6 (2.7) / 108.2 (3.5)	107.1 (2.8) / 108.5 (4.7)	–1.48 (–3.36, 0.41)	0.119
Serum aldosterone, pmol/l	16 / 14	153.3 (162.6) / 214.6 (541.1)	42.7 (34.5) / 182.0 (234.3)	–66.20 (–95.95, –36.44)	<0.001

BL, baseline; CI, confidence interval; EOT, end of treatment; LSM, least squares mean; SD, standard deviation; SZC, sodium zirconium cyclosilicate.

All measures were made as part of pre-defined clinical laboratory safety assessments.

Comparison between groups calculated using an ANCOVA analysis with *P*-values based on *t* test and treatment differences calculated as SZC minus placebo; positive values for comparison between groups signify change for SZC > placebo.
